# Structural Aspects of the Interaction of Dairy Phages with Their Host Bacteria 

**DOI:** 10.3390/v4091410

**Published:** 2012-08-31

**Authors:** Jennifer Mahony, Douwe van Sinderen

**Affiliations:** 1 Department of Microbiology, University College Cork, Western Road, Cork, Ireland; Email: j.mahony@ucc.ie; 2 Alimentary Pharmabiotic Centre, Biosciences Institute, University College Cork, Western Road, Cork, Ireland

**Keywords:** bacteriophage, milk fermentation, receptor

## Abstract

Knowledge of phage-host interactions at a fundamental level is central to the design of rational strategies for the development of phage-resistant strains that may be applied in industrial settings. Phages infecting lactic acid bacteria, in particular *Lactococcus lactis *and *Streptococcus thermophilus*, negatively impact on dairy fermentation processes with serious economic implications. In recent years a wealth of information on structural protein assembly and topology has become available relating to phages infecting *Escherichia coli*, *Bacillus subtilis *and *Lactococcus lactis*, which act as models for structural analyses of dairy phages. In this review, we explore the role of model tailed phages, such as T4 and SPP1, in advancing our knowledge regarding interactions between dairy phages and their hosts. Furthermore, the potential of currently investigated dairy phages to in turn serve as model systems for this particular group of phages is discussed.

## 1. Introduction

Phages are the most abundant biological entities on Earth [1] and are responsible, at least in part, for driving the evolution of their bacterial hosts [2]. The selective pressure imposed by phages on their hosts also requires an equivalent genomic plasticity and adaptability by the phages themselves in order to successfully produce progeny in a host that tries to escape its parasites. All characterized phages known to infect lactic acid bacteria (LAB) possess a tail and therefore this review will solely consider tailed phages. 

One of the first physical phage-host interactions is that between the receptor on the host bacterium and the distal end of the tail of the phage. Many isolated phages have been characterised morphologically by electron microscopy analysis [3] and the information derived from such analyses may be useful to gain an insight into the position and nature of host receptor (see below). In recent years structural information has emerged related to phage proteins that are involved in binding to these receptors, usually referred to as anti-receptors, receptor binding proteins (RBPs) or adhesins. Depending on the macromolecular nature of the receptor, *i.e.*, being a protein or carbohydrate, the phage tail tip of *Siphoviridae* phages, which contains the receptor-binding protein, appears to display a particular morphology. For example, phages that interact with protein moieties generally possess either a pointed or “stubby” end, while those that interact with carbohydrate moieties more often display a larger, more obvious structure, which is called the baseplate. While there are exceptions to this generalisation, including many undefined phage-host interactions, this may perhaps be the primary indicator of the type of moiety required by a phage to interact with its host. Here we will explore the role of electron microscopy and structural analysis in defining the interactions between phages and their hosts, and examine the various types of receptor-binding proteins encoded by phages and their corresponding host-encoded receptor. The interactions and wealth of structural data for phage RBPs that now exists will be examined with a view to understand the interactions between dairy phages and their hosts. Strains of *Lactococcus lactis* and *Streptococcus thermophilus *are the most widely utilised LAB in the dairy industry, with certain species of *Lactobacillus* also being used intensively. Consequently, phages infecting these species dominate LAB phage research. Since the most frequently isolated, and hence the most problematic phages of dairy lactic acid bacteria (LAB) are those belonging to the *Siphoviridae *(isolated for *Lactococcus*, *Streptococcus *and *Lactobacillus *spp., among others) and, to a lesser extent, *Myoviridae* families (isolated for *Lactobacillus *spp.), we will focus on the former for purposes of clarity.

## 2. Phages that Recognize a Proteinaceous Receptor

Phages recognizing a proteinaceous receptor can achieve a strong phage-bacterium interaction through protein-protein interactions and therefore only a single phage protein or small complex is required due to the high affinity, avidity and specificity of this interaction [4]. In electron micrographic images such phages possess a pointed or stubby tail tip, which is where the RBP is located (See Table 1 for examples). A very well established example of this is the *Bacillus subtilis*-infectingsiphophage SPP1, which interacts irreversibly with its host-encoded receptor protein YueB via its pointed tail tip following an initial and reversible attraction to a carbohydrate moiety on the cell surface [4]. Additionally, the lactococcal c2-type siphophages interact with the phage infection protein (PIP) through a RBP that is presumed to be located at the blunt-ended tail tip [5]. It is noteworthy that both YueB and PIP are members of the Type VII secretion system and are closely related proteins and therefore functional analogies are perhaps not surprising. The tail adsorption protein of c2 is proposed to be encoded by orf *l10*, a 75 kDa protein identified through mass spectrophotometry and immune‑gold electron microscopy [6]. The bacterial receptors of the *Siphoviridae* coliphages λ and T5 (both of these phages possess pointed tail tips) have been defined and isolated (LamB and FhuA, respectively), which has permitted the development of *in vitro* DNA ejection assays to positively identify these receptor proteins [7,8]. The coliphages belonging to the *Myoviridae *T4 superfamily possess six long tail fibers that interact reversibly with lipopolysaccharide (LPS) or a porin protein, such as OmpC [9,10]. Subsequently, the baseplate is lowered toward the cell surface and the six short tail fibers are released to allow reversible binding to occur, thereby allowing the pointed puncturing device to initiate DNA injection into the host after the baseplate is switched to the star conformation [11]. Of the above-mentioned phages, T4 is one of the best studied model phages with respect to the structural analysis of the tail fibers and injection devices. The 3D structure of the long tail fiber receptor-binding domain of gp37 has been resolved, which revealed that the aromatic and positive residues on the surface of the fiber tips are most likely receptor-binding determinants with the receptor, although the authors do not specify whether the receptor material is LPS or the OmpC porin protein [9]. The proteins constituting the T4 baseplate complex have been studied by means of cryo‑electron microscopy and X-ray crystallography [11,12,13]. Through these analyses, at least twelve proteins have been identified as baseplate components: gp5, gp6, gp7, gp8, gp9, gp10, gp11, gp12, gp25, gp27, gp53, gp54. The six gp10 trimers are a crucial component of the baseplate and structural analysis of this protein has revealed its role in extension of the short tail fibers encoded by gp12 by means of a partner shift: the pre-infection state sees an interaction between the C-terminal end of gp10 and gp12, while infection requires an interaction between the baseplate protein gp9 and gp10 so as to release the short tail fibers, thus permitting interaction with the host receptor [13]. This fascinating and complex phage serves as an excellent model for the physical interactions between a phage and its host. However, this model is much more sophisticated than many of the phages of the *Siphoviridae* family and for this purpose phage SPP1 may be a better representative for this family of phages.

**Table 1:** Proven and putative phage receptors.

Host	Phage	Receptor type	Host receptor	Reference
*Bacillus subtilis*	SPP1	Protein	YueB	[14]
*Escherichia coli*	T4-like	Protein	OmpC/LPS	[10]
	T5	Protein	FhuA	[8]
	Λ	Protein	LamB	[7]
*Lactococcus lactis*	c2	Protein	PIP	[5]
	p2	Saccharide	Unknown	[15]
	bIL170	Saccharide	Unknown	[16]
	TP901–1	Saccharide	Unknown	[17]
	Tuc2009	Saccharide	Unknown	
*Lactobacillus*	LL-H	Lipoteichoic acid	Poly-Glycerophosphate LTAs	[18]
*Streptococcus thermophilus*	OBJ	Saccharide	Glucosamine/Ribose	[19]
	CYM	Saccharide	Glucosamine/Rhamnose	[19]

## 3. SPP1 as a Model for Protein-Interacting Siphophages of Gram-Positive Bacteria

SPP1 possesses a long non-contractile tail, which is the trademark property of the *Siphoviridae* phages. At the base of the tail is a narrow device, the tail tip, which interacts with a membrane-anchored protein (YueB) on the host cell surface [14]. Significant structural data has emerged in recent years relating to the capsid, tail, head-tail connector and the tail tip of SPP1, thus rendering it a well understood model for other siphophages infecting Gram-positive bacteria, including those that target LAB [20,21,22,23,24,25,26,27].

One of the first structural studies of phage SPP1 focused on the tail at pre- and post-infection stages, and detailed the loss of the tail tip following DNA ejection [14]. The tail of SPP1 is composed of an estimated 40 stacked rings (gp17.1 and gp17.1*), inside which is a channel containing the tail tape measure protein (gp18) and through which the phage genome is passed upon release from the capsid [14]. The outside of the tail rings exhibit a six-fold symmetry (gp17.1 and gp17.1*, see below), while the inner tube has a continuous appearance that is composed of several gp18 subunits [14]. The tail tip, which has no channel to allow DNA passage, interacts with YueB, which prompts its release from the tail cap, thereby triggering a sequence of conformational changes that result in the ejection of the viral genome from the capsid. Following DNA ejection, the internal channel diameter appears to undergo a size reduction from 56 Å to 42 Å due to rearrangements in the major tail proteins gp17.1 and gp17.1* and not negating the fact that the tail tape measure protein fills the channel prior to DNA ejection [14]. These gp17.1 and gp17.1* proteins are present in a 3:1 ratio and protein microsequencing has demonstrated that the product of gp17.1* contains an additional C-terminal 10 kDa region, which is thought to be exposed on the outer surface of the tail [28]. This feature also seems to be encoded by prophages of *Bacillus licheniformis *and *Bacillus halodurans*, while such extensions also appear to be present in the phage receptor-binding proteins and major tail protein‑associated sequences of other *Siphoviridae *phages such as the lactococcal phage Q54 and *Listeria* phage A118 [28].

**Figure 1:**
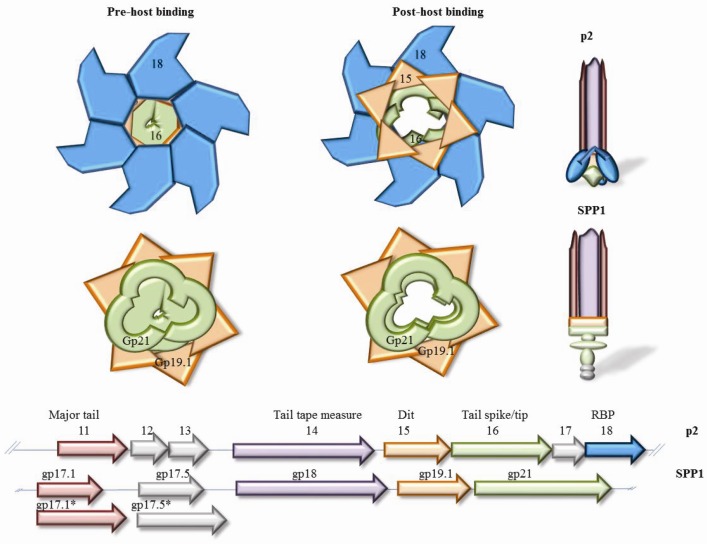
Schematic representation of a top view of the re-organisation of the p2 (top) and SPP1 (bottom) baseplate regions before (right) and after (left) binding to the host cell. The p2 baseplate, composed of a hexamer of ORF15 (orange), a trimer of ORF16 (green) and six trimers of ORF18 (blue) which represents the RBP. In parallel, the SPP1 distal tail region is composed of two back-to-back hexamers of gp19.1 (orange) and a trimer of gp21 (green) bound to each of the Dit (gp19.1) hexamers, one trimer in the open conformation and the other in the closed conformation. A representation of the genomic regions that encode the tail structural components are presented in the lowermost section, including the genes encoding the proposed major tail protein (pink), the tail tape measure protein (purple), the distal tail protein (Dit)/hub protein (orange), the tail spike (SPP1) or tail tip (p2) (green) and the RBP (blue, p2). Those in grey encode non-structural proteins or have not been functionally assigned.

Other elements of the distal end of the tail of SPP1 have been characterised including the distal tail protein (Dit, gp19.1) and the N-terminal region of the tail spike protein (gp21) [23,25]. The SPP1 Dit appeared as a dimer of hexamers in a back-to-back orientation which is structurally similar to a component of the lactococcal phage p2 baseplate (ORF 15), however it is now clear that this apparent dimer is a crystallographic artifact in the case of SPP1 [23]. Two trimers of the SPP1 truncated tail spike protein have been shown to interact with Dit, where one trimer is presumed to be in a “closed” conformation while the other trimer is in the “open” conformation (Figure 1) [25], a system which is consistent with observations made for the lactococcal phage p2 baseplate [25,29]. The Dit-tail spike complex appears to constitute the tail cap of SPP1 and the structural conservation of these features in lactococcal phages may indicate a common lineage of such systems [25]. Calcium ions stabilise the “open” conformation of tail cap, which is also consistent with the lactococcal phage p2 [25,29]. The Dit-tail complex acts as a hub upon which the baseplate (in the case of phages that recognize a saccharidic receptor) or the tail tip/spike (for phages that bind to a proteinaceous receptor) is hinged, at the distal end of the tail. Furthermore, (multimers of) the major tail protein (MTP) and tail tape measure protein (TMP) attach to this structure to form the tail tube. It is suggested that the interaction of the RBP with the host-encoded receptor induces tail base to change to the open conformation triggering the movement of the first ring of MTP which is then sequentially continued up through the MTP rings with ensuing release of DNA from the phage capsid [14,25]. The structural conservation of the Dit-tail cap complex and conformational arrangements in SPP1 and lactococcal phages endorses SPP1 as a model for *Siphoviridae* phages, and as such is a basis for understanding the interactions between dairy phages and their hosts.

## 4. Structural Approaches to Understand Dairy Phage Interactions and Carbohydrate-Recognizing Phages

Lactococcal phages have, among the dairy phages, received the most extensive scientific scrutiny with regards to the structural analysis of protein/protein complexes [17,29,30,31,32]. Isolated lactococcal phages are grouped into ten distinguishable species based on DNA homology and particle morphology [33]. Of these, the 936, c2 and P335 species are most frequently encountered in dairy facilities world-wide. Members of the 936 and P335 species are believed to bind a saccharide component that is present on the surface of the cell envelope, while the c2 phages are known to bind the so-called phage infection protein (PIP) [5]. Members of the 936 and P335 species have been studied at a structural level and constitute the backbone of emerging structural data relating to dairy phages. 

### 4.1. Lactococcal P335 and 936 Phage-Host Interactions

Over the past decade, significant efforts have been made to determine the molecular nature and mechanism of the interactions between lactococcal phages and their hosts, and members of the P335 and 936 species have been central to such studies due to their industrial relevance. It has long been suggested that these phages recognize a carbohydrate moiety on their host, a notion that is consistent with carbohydrate inhibition assays [34]. Through mutational analyses several adjacent genes within an operon, which encodes the biosynthetic machinery for a cell envelope-associated polysaccharide (also called the pellicle), have been shown to be required for adsorption of 936-type phages. The involvement of this pellicle biosynthesis operon in phage infection has been demonstrated in two separate studies for three 936-type phages, namely bIL170, 645 and sk1, although the precise role of the mutated genes as a phage receptor has not yet been established [35,36]. The 20–25 kb pellicle operon contains elements that are conserved among all sequenced lactococcal strains, while it also contains strain-specific regions that are expected to lead to the production of pellicles with a distinct saccharidic structure and composition. The so-called “adsorption genes”, or receptor-encoding genes, involved in 936-type phage recognition and adsorption encode either a glycosyltransferase or a membrane-associated protein [35]. The involvement of these gene-products may imply two possibilities of the mode of interaction and DNA injection pathway of these phages. Firstly, that the glycosyltransferase inactivation may prevent the transfer and decoration of the required pellicle monosaccharide component to the surface thus negating the primary contact between the phage RBP and its saccharide receptor; or secondly, if primary contact is established and a membrane component is required for the translocation of the DNA across the membrane, the membrane-associated protein inactivation prevents internalisation of the phage DNA. Chemical mutagenesis of the lactococcal host strain 3107, which is sensitive to the P335 phages LC3 and TP901–1, resulted in the isolation of five genetically and phenotypically distinct mutants resistant to one or both phages [37]. This analysis revealed that the injection pathways employed by these phages of the same species are distinct. It is through studies such as these that more attention has been diverted towards the identification of phage RBPs on lactococcal phages in order to determine the relationship between a phage and its host(s).

The 936 phage RBPs has been identified through the isolation of a chimeric sk1/bIL170, which also was shown to exhibit a “host-range swapping” phenotype [38]. The identification of the RBPs of these phages presented the opportunity to define the structure of the corresponding proteins and their associated complexes. This study applied an approach previously used by Duplessis and Moineau (2001) in which the variable C-terminal VR2 region of ORF18 of DT1 was replaced by the corresponding fragment of MD4. The resultant chimeric DT1 infected the host for MD4, thereby elucidating the host specificity function of this protein [39]. Similarly, a chimeric derivative of phage TP901–1 (designated TP901–1C), harbouring the lower baseplate protein of phage Tuc2009 instead of its original TP901–1 equivalent, was shown to have acquired the host range of Tuc2009, thereby identifying the lower baseplate protein as the RBP of both of these P335 phages [40]. Such discoveries have been very useful in the assignment of RBPs of other P335 phages through homology and genomic positioning of the encoding gene [41]. Also noteworthy is the observation of the altered baseplate morphology in the case of the chimeric phage TP901–1C as compared to the parent phage [36]. TP901–1 has a clear double-disc baseplate while the chimeric phage TP901–1C was described to possess “hanging droplets” rather than a distinct lower baseplate and furthermore the lower disc appeared narrower than that of the parent phage (23 nm *versus* 28 nm in the parent phage) and intermediate between that of the wild-type TP901–1 and Tuc2009. The genetic plasticity of the lactococcal phages has permitted the development of recombinant phages of both the 936 and P335 species and may present a mechanism by which these phages alter their host specificity or enlarge their host spectrum as their environment dictates.

### 4.2. 936 Phage Baseplates

The first lactococcal phage RBP to be structurally defined was that of the 936 phage p2 [42]. The p2 RBP is a homotrimeric protein comprising three domains: the head, neck and shoulder. The actual receptor binding site was identified within the “head” domain through the application of the heavy chain neutralising llama antibody fragment V_HH_5, which had previously been determined to prevent infection of the lactococcal host by phage p2 [15,42,43]. The shoulder domain possesses eight ß‑strands interspersed with an α-helix between ß-strands one and two, and a coil between ß-strands two and three [27]. The neck is a ß-prism of three segments that yields a rigid structure to which analogy has been drawn to the gp12 short tail fiber structure of the coliphage T4. Finally, the head domain is a ß-barrel of seven anti-parallel ß-strands [42]. Seven charged or polar and five non-polar residues were identified in the RBP head domain interacting with the V_HH_5. 

The receptor binding site was further characterised through the isolation of escape mutants that could bypass the llama neutralising antibody fragments [15]. The majority of the mutations that enable the mutant phages to overcome the llama antibodies are within the interacting site. Just twelve out of fifty assessed lactococcal 936 phages were neutralised by V_HH_5 antibodies, highlighting the apparent variability of RBPs found in members of this closely related phage species. Mutants overcoming the V_HH_5 were readily isolated for eleven of the twelve phages neutralised and in all cases a single point mutation in the corresponding RBP-encoding gene was shown to be sufficient to prevent antibody recognition. This study also served to improve the resolution of the RBP structure of p2 from 2.3 to 1.7 Å and identified a glycerol molecule bound to the structure originating from the cryoprotectant. The binding of the glycerol molecule was observed to be tighter in the head domain than the shoulder domain where three hydrogen bonds are formed between head domain residues and the glycerol O-1 and O-2 atoms (*versus* one in the shoulder domain). Other saccharides were assessed for their ability to bind the RBP in solution and it was suggested that phosphoglycerol, which is a component of lipoteichoic acid and teichoic acid, represented a potential receptor. This was the first direct proof of carbohydrate binding of a lactococcal 936-type phage RBP, confirming biological analyses that suggested the role of specific carbohydrates as receptors for 936-type phages [15,34].

The crystal structure of the RBP head domain of another 936 phage, bIL170, was solved and represents one of a number of phages that were not neutralised by the V_HH_5 antibodies due to its variable C-terminal sequence [16]. The phylogeny of the RBPs of 936 phages has demonstrated clear links to host range and V_HH_5 antibody-neutralisation, and it is well established that while the N‑terminal region is well-conserved, it is the C-terminal (head) domain that determines the specific host interaction [15,45,46,47]. Interestingly, while there is little sequence conservation between the head domains of p2 and bIL170, their structure is strikingly similar [16]. Furthermore, it bears structural similarity to the head domain of the lactococcal P335 phage TP901–1 [16]. Akin to the RBP of p2, the head domain of bIL170 is proposed to be a homotrimer that binds saccharides near a tyrosine residue (Tyr226) whose side chain forms the upper wall of the crevice between the trimer components and is accessible to solvents [16]. 

Expression and purification of ORFs 15, 16, 17 and 18 of p2 by affinity chromatography revealed that the baseplate of p2 is composed of three proteins, ORFs 15, 16 and 18. The structural data generated from crystals, obtained from the *in vitro* expressed recombinant baseplate complex, was mapped onto the baseplate structure, generated from electron microscopy images of the virion, provided comparative data to verify the accuracy of the expressed baseplate complex structure [29]. ORF15 and 16 readily mapped onto the virion structural data while ORF18 displayed some variation. This variation was proven to be the result of conformational swapping of the RBP head domains either upward, facing the capsid in an inactive form when calcium is absent, or in the “activated” form in the presence of calcium (or strontium) in which case the RBP head domains rotate 200° downward. Calcium is required for infection by many phages and therefore it is not surprising that it may be involved in the initial stages of the phage-host interaction, however, the conformational swapping of the RBP head domains is the first report of any such baseplate activation for *Siphoviridae* phages [29]. Such studies highlight the importance of electron microscopy analysis to define the native virion baseplate structures because, while the use of camelid antibodies provided data on the baseplate/RBP in the activated state, the state of the free phage baseplate revealed a previously unknown mechanism. While calcium is known to be required for the activation of the baseplate, it is unclear as yet if there is also as role for calcium in the physiology and organization of the bacterial envelope, thereby endorsing phage infection or if the effect is limited to the activation of the phage baseplate.

The application of these “block cloning” strategies to the expression and purification of baseplate complexes of the lactococcal phage p2 and TP901–1 is an important tool to facilitate the study of lactococcal base plates [47,48]. This block cloning also provides discerning information that may define which proteins of an operon form part of the complex and this approach may be applied to any operon encoding structural components of heteromeric organelles. A model for the construction and assembly of lactococcal P335 phage tails has been proposed in which the tail tape measure (TMP), distal tail protein (Dit) and tail-associated lysin (Tal) converge to act as the “initiation complex” upon which the baseplate is assembled [40,41]. Mass spectrometry has recently been used as an alternative method to define the assembly pathway of the baseplates of p2 and TP901–1 [49]. Using this approach, the p2 baseplate is proposed to assemble through the interaction of a hexamer of ORF15 with a preassembled ORF16 trimer followed by the capture of six ORF18 trimers [49]. This approach was also applied to the P335 phage TP901–1 and an assembly pathway was proposed which is outlined below.

### 4.3. P335 Phage Baseplates

**Figure 2:**
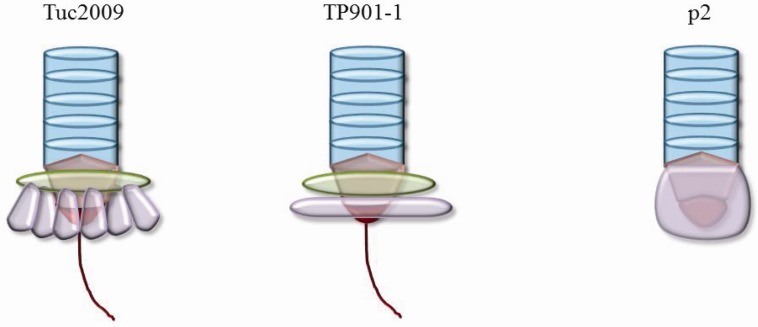
A schematic representation of the distal tail region of Tuc2009, TP901–1 and p2. The stacks of tail rings composed of the major tail protein (MTP) (blue) with the distal tail (Dit) protein (orange) beneath, upon which the baseplate components are hinged (green and purple). In the case of Tuc2009 the green area represents the upper baseplate disc (BppU and BppA) while the TP901–1 upper disc is composed of BppU only. The lower baseplate (BppL) is presented in purple with a disc representation for TP901–1 and a so‑called “petticoat” representation for Tuc2009. The protrusion from the baseplates of TP901–1 and Tuc2009 represent the tail associated lysin (Tal) (red). The schematic of the baseplate region of p2 highlights ORF15 (orange) at the base of the tail with ORF16 beneath (red) and the RBP in the active downward orientation (purple).

The possible assembly pathway for TP901–1 that was proposed involves the interaction of homotrimers of ORF48 (upper baseplate protein) and ORF49 (lower baseplate protein) [49]. These then form tripods comprised of 3×ORF48 and 9×ORF49, and after interaction between ORF48 with ORF46 (distal tail protein or Dit), oligomerisation of ORF46 may proceed with concomitant baseplate formation with a proposed final composition of 6×ORF46, 18×ORF48 and 54×ORF49 [49]. Furthermore, through electron microscopic analysis of the TP901–1 baseplate together with that of TP901–1 mutants lacking either BppU (upper baseplate) or BppL (lower baseplate), the structure and composition of the TP901–1 baseplate have been determined [31]. Structural analysis of the RBP of TP901–1 has revealed the similarity of the modularity of the RBP of TP901–1 and the previously studied p2, pointing to common ancestral origins of such structures (Figure 2) [17]. This is consistent with the development of a chimeric RBP with the N-terminal portion of TP901–1 and the C-terminal head domain of p2 [50]. The resulting structure presented domains almost indistinguishable from the parental structures highlighting the modular conservation of these lactococcal phages of distinct species [49]. The head domain of TP901–1 RBP was also observed to bind glycerol and fluorescence quenching further consolidated the carbohydrate affinity of this phage protein, identifying affinity for glycerol and muramyl-dipeptide [17]. 

Unlike the 936-type phage p2, the TP901–1 baseplate is maintained in a so-called “infection-ready conformation” [31,51]. This observation highlights the bipartite baseplate conformations either maintained in an infection-ready state or in a state requiring activation. It is suggested that the signalling to induce DNA ejection after adsorption of TP901–1 may require a more subtle conformational change than that of p2 and the T4 Myoviruses [51]. In this study, two potential infection pathway scenarios were proposed: first is the possibility that the tail-associated lysin (Tal) may interact with the cell wall peptidoglycan resulting in triggering of DNA release through a so‑called cascade effect channelled up through the tail tube in a similar manner proposed for SPP1 [14,51]. The second proposed injection pathway involves the binding of the RBP tripod complexes to the carbohydrate moiety on the cell surface with mechanical alterations leading to signalling to the lowermost ring of the major tail protein with ensuing “un-screwing” of the tail to allow DNA ejection to occur [51]. Furthermore, comparisons have been drawn between the activation systems of p2 and the Myophage T4 upon interaction with a carbohydrate moiety, which is an interesting analogy and further corroborates theories of common ancestral elements and conservation of systems and modular structures in the case of RBPs [46]. 

Tuc2009 is a temperate P335 phage that is closely related to TP901–1 [52], although its baseplate‑encoding operon contains an additional gene designated *bppA* (accessory baseplate protein). This appears to be a non-essential structural element of the Tuc2009 baseplate that is possibly involved in the determination or extension of host range. A low resolution model of the Tuc2009 baseplate has been determined in which the baseplate was proposed to consist of six tripods, each comprising 3×BppU, 3×BppA and 3×BppL, although this model is currently being refined that might align Tuc2009 more closely to TP901–1 in terms of its baseplate structure [30]. Structural analysis of this baseplate may uncover a potential role for BppA, for which a functional assignment remains to be provided. The presence of homologues of BppA in other lactococcal P335 phages (ul36 ORF303, accession no. NP_663688.1; P335 ORF45, accession no. ABI54248.1), indicates that there may be a biological significance and ecological advantage to phages possessing the protein in their baseplate. Furthermore, the recent finding that Tuc2009 is one of the few sequenced members of the P335 species that requires calcium to produce plaques may highlight the subtleties that distinguish individual phages within a species [51]. Although significant data is emerging relating to the structure of P335 phage baseplates and individual components thereof, there remains a large gap between our knowledge of these phages and those of the model phages T4 and SPP1. While analogies may be drawn between these models and dairy phages it is imperative to also define relevant structural models within the dairy phage group.

## 5. Conclusions and Future Perspectives

Structural studies relating to T4 in the 1990’s and early 2000’s paved the way for the structural analyses of many phage receptor binding proteins and their associated complexes and have permitted the detailed analysis of several phage-host interactions [9,10,11,12]. This, together with the isolation of the receptor for T5 and lambda in the 1990’s and the 1970’s prompted some of the early phage-host interaction studies for lactococcal phages [7,8,34]. Since then, numerous studies relating to the interactions of dairy phages and their hosts have been reported and have led to a surge of interest in structural analyses of these systems. The lactococcal phages p2, TP901–1 and Tuc2009 are emerging as model phages for the dairy *Siphoviridae* phages in terms of structural analyses. These data present a fresh perspective on studies attempting to define the host molecules with which dairy phages interact. The type of baseplate as defined by electron microscopy may be the initial indicator of the material that acts as receptor for the individual phage although this is merely a first glimpse. Structural analysis and/or detailed electron microscopic analysis may provide detailed information on the baseplate organisation and structure, which may be applied to phage-host interaction studies. The analysis of such structures is paramount to understanding how dairy phages bind to and subsequently infect their hosts, and an improved knowledge of phage-host interactions will aid in developing novel and effective anti-phage strategies such as those already explored through the use of camelid antibodies and designed ankyrin repeat proteins (DARPins) to inactivate phages [15,32]. 

Since structural data relating to these model dairy phages continue to accrue, it is inevitable that these will become the dominant model for the analysis of other dairy phages including those of *S. thermophilus *and *Lb. delbrueckii*, typical cultures used in dairy fermentations. It has been suggested that the receptor material for the *Lb. delbrueckii* phage LL-H is polyglycerophosphate type lipoteichoic acids [18], which is reminiscent of affinity of the 936 phage p2, further demonstrating the usefulness of the lactococcal phage data as a suitable model for other dairy phages. However, while structural analyses are essential to furthering our knowledge of phage-host interactions, it is equally important to apply biological and functional characterisation to verify conclusions drawn from structural analyses. The marriage of molecular microbiology with structural biology represents one of the most powerful modern tools to understand complex interactions and pathways involved in host recognition and infection, and may be the key to developing next generation anti-phage systems with potential application in the dairy industry.
